# Baicalein induces apoptosis in esophageal squamous cell carcinoma cells through modulation of the PI3K/Akt pathway

**DOI:** 10.3892/ol.2012.1069

**Published:** 2012-12-10

**Authors:** HONG-BO ZHANG, PING LU, QING-YIN GUO, ZHEN-HUA ZHANG, XIANG-YU MENG

**Affiliations:** 1 Department of Anatomy and Cell Biology, School of Basic Medicine, Zhengzhou University, Zhengzhou 450001;; 2 School of Pharmacy and Henan University of Traditional Chinese Medicine, Zhengzhou 450008, P.R. China; 3 First Clinical College, Henan University of Traditional Chinese Medicine, Zhengzhou 450008, P.R. China

**Keywords:** cell apoptosis, baicalein, esophageal squamous cell carcinoma, PI3K/Akt signaling pathway

## Abstract

Baicalein, a flavone present in *Scutellaria baicalensis* Georgi, has been demonstrated to possess antitumor activity in a variety of cancer cells *in vitro*. However, its effects on the growth inhibition and induction of apoptosis in human esophageal carcinoma cells remain unclear. The aims of this study were to determine whether cultured EC-109 esophageal squamous cell carcinoma (ESCC) cells undergo apoptosis when treated with baicalein and to investigate the underlying mechanisms *in vitro*. Cell growth was measured using MTT and plate colony formation assays. Induction of apoptosis was examined using Hoechst 33258 staining, flow cytometry analysis and a DNA fragmentation assay. The mechanisms underlying the observed growth suppression were examined using western blot analysis. The results demonstrated that treatment of EC-109 cells with baicalein for 48 h markedly decreased the rate of cell viability. Colony formation was almost fully suppressed at 40 *μ*M baicalein. EC-109 cells underwent apoptosis in response to baicalein treatment, demonstrated by an increase in the percentage of cells stainable with Hoechst 33258 and Annexin V-FITC/PI, increased DNA fragmentation and activation of the intrinsic (mitochondrial) pathway for cell death. The latter was characterized by increased expression of the cleaved forms of caspase-9 and -3, and poly (ADP-ribose) polymerase (PARP). Additionally, baicalein was found to downregulate anti-apoptotic components and upregulate apoptotic components of the PI3K/Akt pathway. In conclusion, baicalein induces apoptosis in EC-109 cells through modulation of the PI3K/Akt pathway, thus providing further understanding of the molecular mechanisms of baicalein action in esophageal carcinoma. Therefore, the present study revealed that baicalein significantly inhibits growth and induces apoptosis in EC-109 human ESCC cells *in vitro*.

## Introduction

Esophageal cancer is one of the most aggressive human cancers. It is currently the sixth leading cause of cancer-related mortality worldwide (1,2). Cancer of the esophagus is associated with a very poor survival rate. Even in the most developed countries, the 5-year survival rate ranges merely from 10–16% (3). In China, the mortality rate of esophageal cancer is ranked fourth among all cancer-related mortalities, and esophageal squamous cell carcinoma (ESCC) is the major histological type (4,5). Despite significant advances in screening, surgical care and chemoradiotherapy techniques, the prognosis for patients with ESCC remains poor (6,7). Thus, it is necessary to search for new treatment strategies.

Numerous Chinese herbs have been discovered to be potential sources of antitumor drugs (8,9). Baicalein (5,6,7-trihydroxyflavone) is one of the key flavones present in *Scutellaria baicalensis* Georgi, which has been used for the treatment of inflammation, cardiovascular disease and microbial infections (10–12). Accumulating evidence has demonstrated the antitumor activity of this flavone in a variety of human cancer cell lines (13–16). The molecular mechanisms underlying these effects are speculated to include the modulation of several classes of cyclin-dependent kinases (CDKs) and CDK regulatory subunits (cyclins) to inhibit cell cylce progression (16,17), suppression of proliferation and induction of apoptosis (via activation of the mitochondrial pathway and DNA fragmentation) in malignant cells (14,16,18–20).

The PI3K/Akt pathway plays a critical role in mammalian cell survival and resistance to apoptosis. Alterations in the PI3K/Akt signaling pathway have also been implicated in the occurrence and development of human cancer (21,22). Activation of the PI3K/Akt pathway has been demonstrated to promote survival of esophageal cancer cells *in vitro*, as well as tumorigenicity and metastasis of human esophageal cancer *in vivo*(23–25). In addition, it has been demonstrated that the expression of cell proliferation and cell cycle-related proteins (such as cyclin D1 and p27), as well as cell apoptosis-related proteins (including Bcl-2 and Bax), as the downstream targets of the PI3K/Akt pathway, were regulated by the PI3K/Akt pathway in human ESCC cells (26).

Notably, baicalein-induced apoptosis and proliferation retardation has been demonstrated to be mediated by down-regulation of the PI3K/Akt pathway in human epidermoid carcinoma (27) and bladder cancer (17) cells. However, no studies thus far have examined the effects of proliferation inhibition and induced apoptosis of baicalein on esophageal carcinoma cells. Therefore, we conducted an investigation to ascertain whether baicalein was capable of downregulating the PI3K/Akt pathway in ESCC EC-109 cells concurrently with induction of apoptotic cell death. To our knowledge, the present study provides the first direct evidence that baicalein induces apoptosis in ESCC cells, and that the underlying mechanism may be activation of the PI3K/Akt signaling pathway.

## Materials and methods

### Chemicals and reagents

RPMI-1640 medium, fetal bovine serum (FBS), penicillin G and streptomycin were obtained from Invitrogen Life Technologies (Carlsbad, CA, USA). Dimethyl sulfoxide (DMSO), ribonuclease A (RNase A), Annexin V-Fluorescein Isothiocyanate (FITC) Apoptosis Detection kit, 3-(4,5-dimethylthiazol-2-yl)-2,5-diphenyltetrazolium bromide (MTT) and baicalein (C_15_H_10_O_5_, MW 270.24) were purchased from Sigma-Aldrich (St. Louis, MO, USA). All antibodies (mouse antibodies specific for β-actin, procaspase-9 and -3, cleaved caspase-9 and -3, PARP, Bcl-2, Bax, Akt, p-Akt, NF-κB, IκB, p-IκB, mTOR and p-mTOR) and horseradish peroxidase-conjugated goat anti-mouse secondary antibodies were purchased from Santa Cruz Biotechnology, Inc. (Santa Cruz, CA, USA). Baicalein was dissolved in DMSO. The final DMSO concentration was <1‰ (v/v) in all experiments.

### Cell culture

Human ESCC EC-109 cell line was obtained from the China Center for Type Culture Collection (CCTCC; Wuhan, China). Cultures were maintained in RPMI-1640 medium supplemented with 10% FBS and antibiotics (100 U/ml penicillin and 100 *μ*g/ml streptomycin) at 37°C in a humidified atmosphere containing 5% CO_2_. The study was approved by the Ethics Committee of Zhengzhou University, Zhengzhou, China.

### Examination of morphological changes by a phase-contrast microscopic study

EC-109 cells (2×10^5^ cells/well) were maintained in 12-well plates for 24 h and treated with various concentrations of baicalein (0, 10, 20 and 40 *μ*M) for 24 h. Morphological changes in cells due to each treatment procedure were observed and photographed under a phase-contrast microscope.

### Cell viability assay

Proliferation of cells was determined by an MTT assay. Approximately 10,000 EC-109 cells/well were plated in 96-well plates. Following incubation overnight, cells were treated with baicalein (0, 10, 20 and 40 *μ*M). At various time points (24–72 h) following baicalein treatment, the medium was removed and MTT (20 *μ*l of 5 mg/ml) was added to each well and incubated at 37°C for 4 h. The plates were spun and the purple precipitates of formazan were dissolved in 150 *μ*l DMSO. Absorbance was measured at 490 nm using an enzyme-linked immunosorbant asssay (ELISA) plate reader. The viability of baicalein-treated EC-109 cells was expressed as a percentage relative to non-baicalein-treated control cells. Control cells were considered to be 100% viable.

### Plate colony forming assay

Suspensions of EC-109 cells were inoculated in 6-well flat-bottomed plates with a density of 3×10^2^ cells/well and 3 wells/group. Cells were dispersed evenly by slightly shaking the plates and were then incubated with baicalein at different concentrations in RPMI-1640 medium, with 10% FBS at 37°C and 5% CO_2_ for 14 days, until the visible clones appeared. The medium was discarded and cells were carefully washed twice with PBS. Following fixation with methanol for 15 min, cells were stained with Giemsa’s solution for 15 min before washing with tap water and air-drying. Clones with >50 cells were counted with an ordinary optical microscope. All experiments were repeated in triplicate and the average values are presented.

### Hoechst 33258 staining

Following treatment with baicalein at various concentrations for 48 h, cells were washed twice with PBS and fixed in 1 ml of 4% paraformaldehyde for 10 min at 4°C. After washing twice with PBS, cells were stained with 100 *μ*l Hoechst 33258 in PBS for 15 min at room temperature in the dark, and then washed with PBS. Cells were mounted and examined by fluorescence microscopy (Olympus BX-51, Tokyo, Japan). Apoptotic cells were identified by the condensation and fragmentation of their nuclei.

### DNA fragmentation assay

Following exposure to various concentrations of baicalein for 48 h, EC-109 cells were collected by centrifugation and washed twice with PBS. Cell pellets were resuspended in 40 *μ*l of lysis buffer (0.1 M EDTA; 0.1 M Tris-HCl, pH 8.0 and 0.8% SDS) and subsequently treated with 10 *μ*l RNase A (50 *μ*g/ml) at 37°C for 1 h, and with 10 *μ*l proteinase K (20 *μ*g/ml) at 50°C overnight. Extracted cellular DNA was subjected to agarose gel (2.0%) chromatography at 35 V for 3 h. Gels were photographed following staining with 0.5 *μ*g/ml ethidium bromide.

### Measurements of cells in early and late apoptosis

The ability of baicalein to induce apoptosis in EC-109 cells was examined by Annexin V-FITC/propidium iodide (PI) double-staining and flow cytometry. Preparations were treated with baicalein at various concentrations for 48 h. Cells were then harvested, resuspended to 5×10^5^/ml in binding buffer (10 mM HEPES, pH 7.4; 150 mM NaCl; 5 mM KCl; 1 mM MgCl_2_ and 1.8 mM CaCl_2_), and doubly stained with Annexin V-FITC/PI according to the manufacturer’s instructions. The percentages of viable, early apoptotic, late apoptotic and necrotic cells were determined using a FACSort flow cytometer (Becton Dickinson, San José, CA, USA).

### Western blot analysis

EC-109 cells were treated with 20 *μ*M baicalein for 0–72 h. Then, cells were harvested and lysed on ice for 30 min in lysis buffer containing 50 mM Tris-HCl, pH 8.0; 150 mM NaCl; 20 mM EDTA; 50 mM NaF; 1% NP-40 and 0.02% NaN_3_. The lysis buffer also contained protease inhibitors (1 mM PMSF and 1 *μ*g/ml aprotinin) to prevent proteolysis and/or dephosphorylation. Lysates were collected following centrifugation at 12,000 rpm for 20 min at 4°C. Protein levels were quantified using the Lowry method (28). Equivalent weights of protein (50 *μ*g/lane) were separated by 15% sodium dodecyl sulfate polyacrylamide gel electrophoresis (SDS-PAGE) and transferred to polyvinylidine fluoride (PVDF) membranes. Membranes were blocked with 5% non-fat milk in TBST buffer for 1 h and then incubated with primary antibodies at 1:1000 dilution in 5% non-fat milk overnight at 4°C. Membranes were then washed twice and incubated with secondary antibodies conjugated with horseradish peroxidase at 1:1,000 dilution for 1 h at room temperature. After extensive washing with TBST, protein bands were visualized by the enhanced chemiluminescence reagent (Amersham Pharmacia Biotech, Tokyo, Japan). The relative expression ratios of experimentals and controls were calculated according to the reference band of β-actin by the density using the software Un-SCAN-IT gel Version 6.1 (Silk Scientific, Inc., Orem, UT, USA). Experiments were repeated in triplicate.

### Statistical analysis

Data are expressed as the mean ± standard deviation. Significance of the difference in means between groups was obtained by analysis of variance (ANOVA). P<0.05 was considered to indicate a statistically significant difference.

## Results

### Inhibition of cell growth by baicalein

**Figure 1.**
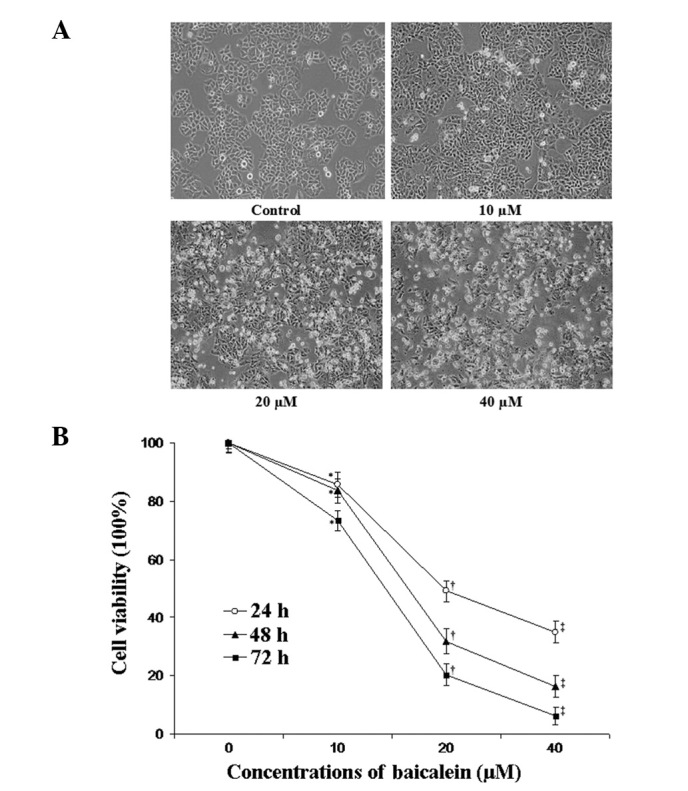
Morphological changes and cell viability of EC-109 cells following treatment with baicalein. (A) Cells after treatment with 0, 10, 20 and 40 *μ*M baicalein for 24 h. (B) Concentration- and time-dependent effects of baicalein on EC-109 cell viability after cells were cultured with various concentrations of baicalein for 24, 48 and 72 h are demonstrated. Findings are presented as the mean ± standard deviation for three independent experiments. ^*^P<0.05 compared with the solvent control; ^†^P<0.05 compared with 10 *μ*M baicalein; ^‡^P<0.05 compared with 20 *μ*M baicalein.

Cell morphology was examined using phase-contrast microscopy. Microscopic observations revealed that EC-109 cells exposed to various concentrations of baicalein underwent significant morphological alterations (Fig. 1A). When exposed to baicalein at a concentration of 10 *μ*M, EC-109 cells began to shrink and retract from the neighboring cells. At a concentration of 20 *μ*M, floating cells began to appear in the culture medium. EC-109 cells incubated in concentrations of 40 *μ*M of baicalein lost their original morphological (flat, polygonal) shape and additional floating cells appeared. An MTT assay was implemented to examine the viability of EC-109 cells exposed to 0, 10, 20 and 40 *μ*M of baicalein in the culture medium for 24, 48 and 72 h. It was found that baicalein significantly decreased the cell viability of EC-109 cells in a concentration- and time-dependent manner (Fig. 1B).

### Inhibition of colony formation

**Figure 2.**
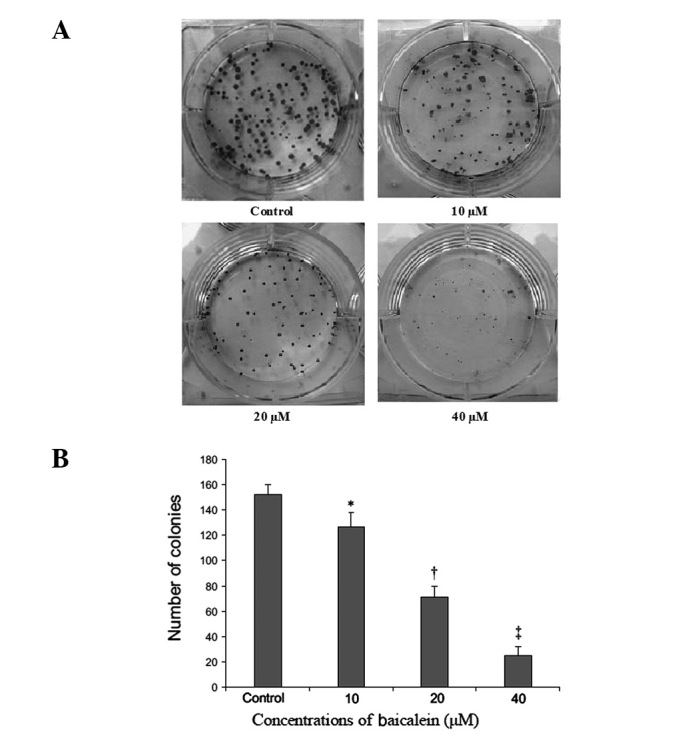
Inhibition of colony formation by baicalein. (A) Colony formation of EC-109 cells following treatment with baicalein at varying concentrations. (B) The number of colonies. Findings are presented as mean ± standard deviation for three independent experiments. ^*^P<0.01 compared with the solvent control; ^†^P<0.01 compared with 10 *μ*M baicalein; ^‡^P<0.01 compared with 20 *μ*M baicalein.

Baicalein also suppressed plate colony formation of EC-109 cells at 14 days post-seeding (Fig. 2A). The number of colonies for control preparations was 152±5.1. By contrast, the number of colonies that formed for preparations treated with baicalein at 10, 20 and 40 *μ*M were 127±4.5, 71±9.2 and 25± 4.5, respectively (P<0.05) (Fig. 2B).

### Baicalein induces apoptosis in EC-109 cells

**Figure 3.**
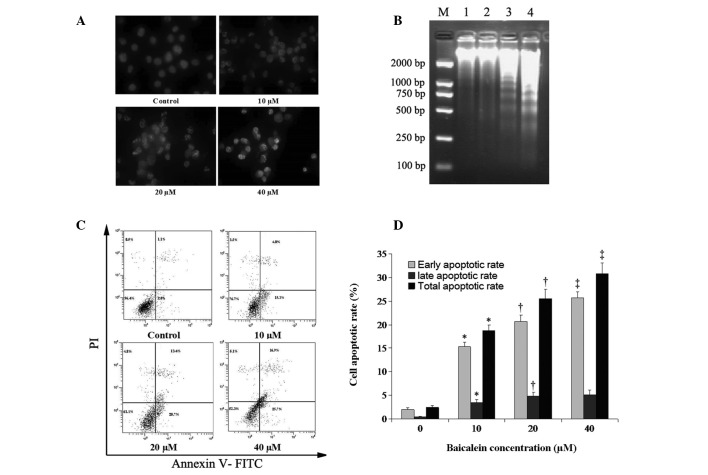
Induction of apoptosis in EC-109 cells by baicalein. (A) Hoechst 33258 staining. The impact of cell apoptosis of baicalein on cultured EC-109 cells without baicalein or following treatment with baicalein at 10, 20 and 40 *μ*M for 48 h. (B) EC-109 cells were treated for 48 h with baicalein at 0 (lane 1), 10 (lane 2), 20 (lane 3), and 40 *μ*M (lane 4). Lane M shows the migration of D2000 markers (100, 250, 500, 750, 1,000 and 2,000 bp). (C) Annexin V-FITC/PI double staining and flow cytometry were used to determine the percentages of cells in apoptosis. Viable, early apoptotic, late apoptotic and necrotic cells were determined following treatment for 48 h with baicalein at various concentrations. Bottom left quadrants, viable cells; bottom right quadrants, early apoptotic cells; top right quadrants, late apoptotic cells; top left quadrants, necrotic cells. (D) Percentages of cells in apoptosis at each baicalein concentration. Cells in the bottom and top right quadrants were summed to obtain the total number of apoptotic cells. Findings are presented as the mean of three similar experiments ± standard deviation. ^*^P<0.05 compared with the solvent control; ^†^P<0.05 compared with 10 *μ*M baicalein; ^‡^P<0.05 compared with 20 *μ*M baicalein.

Hoechst 33258 stain is sensitive to DNA and is used to assess changes in nuclear morphology. The Hoechst 33258 staining assay revealed that cells demonstrated apoptotic features including nuclear shrinkage and chromatin condensation or fragmentation, following treatment with baicalein for 48 h. The rate of cells with a profile of chromatin condensation and fragmented fluorescent nuclei increased in a concentration-dependent manner (Fig. 3A). The ability of baicalein to induce DNA fragmentation, a hallmark of apoptosis, was examined after 48 h of culture. No significant fragmentation was observed in preparations treated with either solvent or 10 *μ*M baicalein. However, fragmentation was clearly observable in preparations treated with 20 and 40 *μ*M baicalein (Fig. 3B). An annexin V-FITC and PI double-staining technique was implemented to investigate whether baicalein induced apoptosis in these cells. The percentage of EC-109 cells undergoing early apoptotic cell death was increased by baicalein in a concentration-dependent manner (Fig. 3C). The percentage of cells undergoing apoptosis was determined by the sum of cells in early and late apoptosis. After 48 h of treatment, 2.5±0.35, 18.8±1.15, 25.5±1.99 and 30.8±2.25% of cells were apoptotic at concentrations of 0, 10, 20 and 40 *μ*M of baicalein, respectively (Fig. 3D).

### Activation of the intrinsic mitochondrial apoptotic pathway

**Figure 4.**
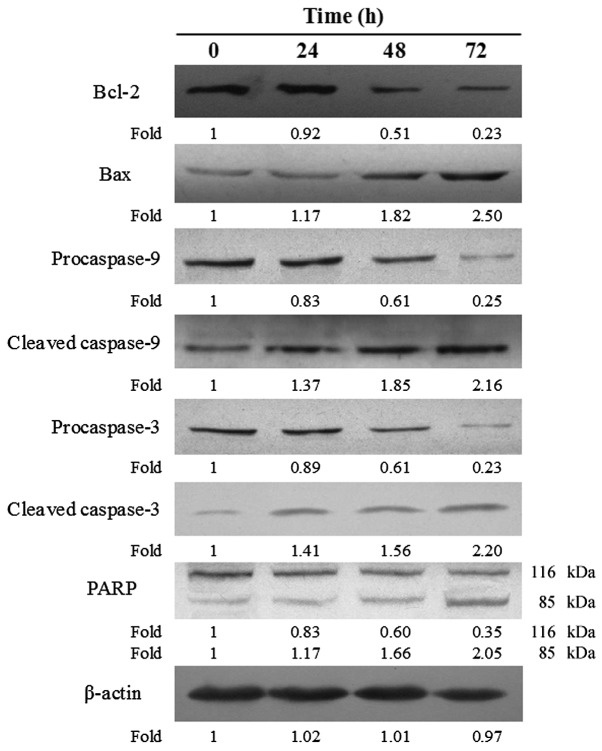
Apoptosis induction via intrinsic apoptotic pathways over time. EC-109 cells were treated with 20 *μ*M baicalein for the times indicated. Expression of β-actin, Bcl-2, Bax, procaspase-9, cleaved caspase-9, procaspase-3, cleaved caspase-3, uncleaved (116 kDa) and cleaved (85 kDa) PARP was analyzed by western blot analysis and the relative ratio was calculated by the density.

It was considered essential to ascertain whether baicalein suppressed the viability of EC-109 cells and promoted DNA fragmentation in such cells through activation of the intrinsic (mitochondrial) apoptotic pathway. Therefore, expression of the relevant apoptosis-related proteins was examined by western blot analysis. Treatment with baicalein increased the expression of pro-apoptotic proteins including Bax, activated (cleaved) caspase-3 and -9, and activated (cleaved) PARP. By contrast, expression of the anti-apoptotic protein Bcl-2, procaspase-3 and -9, and of the inactive form of PARP, was decreased following treatment with the drug. The relative expression ratios of these proteins following baicalein treatment was quantified by the density, and findings are presented in Fig. 4.

### Suppression of the PI3K/Akt pathway

**Figure 5.**
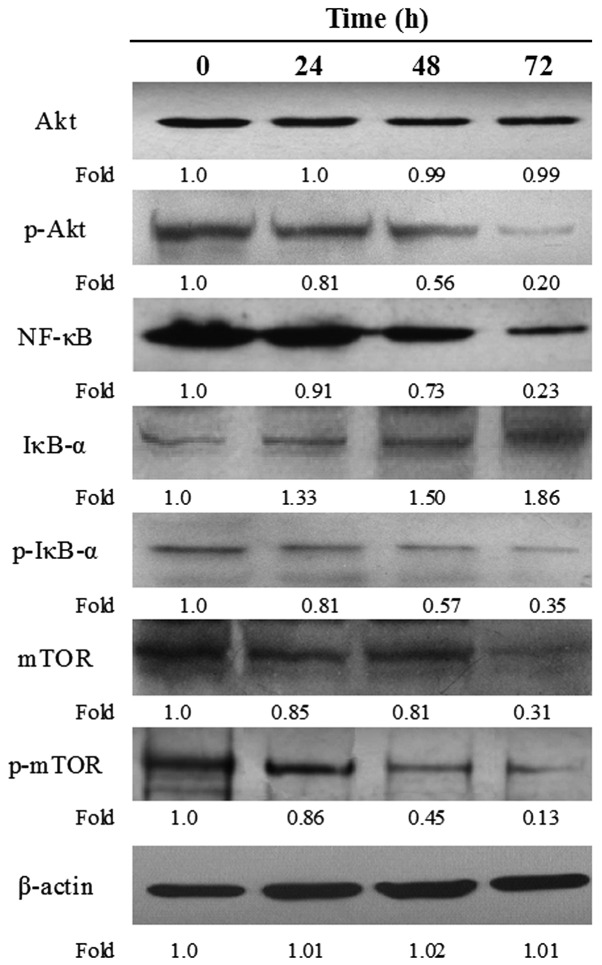
Expression of proteins associated with the PI3K/Akt signaling following various treatment times of EC-109 cells with 20 *μ*M baicalein. Expression of β-actin, Akt, p-Akt, NF-κB, IκB, p-IκB, mTOR and p-mTOR was analyzed by western blot analysis and the relative ratio was calculated by the density.

The effects of baicalein on suppression of the Akt pathway in EC-109 cells were examined. Expression of the following components in their various forms were measured: i) Akt (inactive) and p-Akt (the activated form of Akt); ii) the transcription factor NF-κB, the NF-κB inhibitor, IκB, and the degradable form of IκB, p-IκB; iii) the cell cycle regulatory kinase mTOR (inactive) and p-mTOR, the phosphorylated and active form of the kinase. A decrease in p-Akt and p-mTOR was observed at 24 h of baicalein treatment. Dramatic reductions in the expression of NF-κB and p-IκB were observed in response to baicalein at 48 h of baicalein treatment. These reductions were time- dependent. By contrast, expression of IκB increased with the time of baicalein treatment. The marked decreases in expression of total cellular NF-κB and p-IκB, accompanied by significant increases in IκB expression, in response to baicalein treatment, were interpreted to indicate a condition wherein nuclear NF-κB signaling was dramatically impaired (Fig. 5).

## Discussion

The current study has demonstrated that baicalein is toxic to esophageal carcinoma cells in culture. Treatment with this flavone resulted in a marked decrease in the viability of cultured EC-109 cells and in the number of colonies that these cells formed. Baicalein treatment caused EC-109 cells to undergo apoptosis, as demonstrated by changes in nuclear morphology, an increase in the percentage of Annexin V-stainable cells and an increase in DNA fragmentation.

The PI3K/Akt growth signaling pathway comprises a series of serine/threonine kinase cascades that regulate a variety of cellular processes including cell cycle progression, cell survival and migration, and protein synthesis. Activated Akt acts to phosphorylate Bad, Bax and caspase-9 or activate the NF-κB pathway to promote the resistance of cancer cells to apoptosis (29–32). In the present study, inhibition of Akt phosphorylation, as opposed to downregulation of Akt expression, was observed during treatment of EC-109 cells with baicalein. As Akt acts early on in the PI3K/Akt signaling pathway, it is conceivable that the growth-suppressive effects of baicalein in EC-109 cells are attributable to an interaction of the drug with the kinase. Due to the fact that PI3K expression/activity was not measured in the present study, the involvement of this kinase in the observed effects of baicalein remains unclear. Future studies with various esophageal carcinoma cells lines have been planned to explore the possibility that PI3K is targeted by baicalein.

NF-κB is a critical transcription factor in a variety of physiological and pathological processes. One particular function of NF-κB is to promote cell survival through the induction of target genes, whose products inhibit components of the apoptotic machinery in normal and cancerous cells (33). It has been demonstrated that NF-κB plays a key role in progression, apoptosis and lymph node metastasis in ESCC (34–36). As a downstream component of the PI3K/Akt pathway (37), NF-κB primarily resides in the cytosol in an inactive form; a heterodimer composed of p50 and p65 through an interaction with the inhibitory protein IκB (38). Agonist-induced stimulation resulted in phosphorylation and subsequent proteosomal degradation of IκB, thereby inducing nuclear translocation of NF-κB to promote the transcription of its target genes (38). Activated Akt indirectly signals IκB phosphorylation, thereby promoting transcription of anti-apoptotic genes, whereas inactivation of Akt promotes apoptosis. The present study demonstrated that treatment of EC-109 cells with baicalein induced apoptosis by suppressing components of the PI3K/Akt/NF-κB signaling pathway; the expression of p-Akt, NF-κB, and p-IκB. These decreases were observed concurrently with increased expression of non-phosphorylated IκB.

mTOR, a major downstream target of the PI3K/Akt pathway, is an essential regulator of cell proliferation, protein synthesis and the modulation of signals in various signaling pathways (39,40). The mTOR pathway has a central role in cell growth, as well as in invasion and metastasis of tumors (41–43). It has been demonstrated that the mTOR/p70S6K pathway was activated in ESCC, and rapamycin or siRNA against mTOR rapidly inhibited expression of mTOR, arrested cells in the G0/G1 phase and induced apoptosis of ESCC cells (44). In the present study, the results revealed that a decrease in the expression of mTOR and p-mTOR along with downregulated expression of p-Akt upon baicalein treatement, corroborated well with the growth inhibition and induction of apoptosis in EC-109 cells.

Suppression of Akt in cancer cells is associated with activation of the mitochondrial apoptotic pathway involving the caspase-9-dependent caspase cascade (45). Caspase-3, a downstream caspase, is activated by caspase-9. Caspase-3 activation leads to cleavage and inactivation of key cellular proteins such as PARP and the DNA fragmentation factor (46). In our study, treatment of EC-109 cells with baicalein was revealed to increase the level of cleaved caspase-9 concurrently with a decrease in procaspase-9 protein, to increase level of cleaved caspase-3 concurrently with a decrease in procaspase-3 protein, to increase expression of cleaved PARP concurrently with decreased expression of uncleaved PARP, and to promote DNA degradation. These findings support the hypothesis that apoptotic death in baicalein-treated EC-109 cells is mediated by the following events in sequence: Cleavage of procaspase-9, cleavage of procaspase-3, cleavage of PARP and ultimately degradation of DNA. To further delineate the signaling events involved in baicalein induced apoptosis, the potential role of baicalein in regulating Bax and Bcl-2 proteins, which regulate the essential change in mitochondrial membrane permeability for apoptosis, was examined (47). As a result, baicalein markedly decreased anti-apoptotic Bcl-2 protein levels, while the levels of pro- apoptotic Bax protein concomitantly increased. From these data, it may be concluded that baicalein induced EC-109 cell apoptosis by activating the intrinsic mitochondrial pathway.

In summary, the results of the present study indicate that baicalein induced apoptosis in human ESCC EC-109 cells. This is mediated through suppression of the PI3K/Akt/NF-κB and PI3K/Akt/mTOR signaling pathways which involves activation of the mitochondrial death pathway components (including Bcl-2 family proteins), as well as activation of caspase-3 and PARP. The results of the present study suggest that baicalein may be an effective chemopreventive agent for ESCC. However, further studies are required to determine the upstream signaling that is involved in the inhibition of this signaling pathway by baicalein in ESCC cells. Furthermore, to delineate the potential of this agent in esophageal cancer treatment, we intend to examine the antitumor effects of baicalein in an animal model.
